# A Gas Chromatography‐Based Workflow Leads to the Detection of Hydrogenase Activity in Native Bacteria

**DOI:** 10.1002/cbic.70545

**Published:** 2026-09-30

**Authors:** Marius Stoeckle, Kersten S. Rabe, Christof M. Niemeyer

**Affiliations:** ^1^ Institute for Biological Interfaces 1 (IBG1) Karlsruhe Institute of Technology (KIT) Eggenstein‐Leopoldshafen Germany

**Keywords:** biocatalysis, enzyme activity assay, gas chromatography, hydrogenase, oxygen tolerance

## Abstract

Functional assessment of hydrogenase activity in native bacterial systems is often complicated by the requirements associated with enzyme purification, heterologous expression, and cofactor maturation. Here, we present a standardized gas chromatography‐based workflow for the direct assessment of hydrogenase activity in crude membrane and lysate fractions without enzyme purification or genetic manipulation. The workflow was applied to a defined panel of phylogenetically distinct bacterial strains. Hydrogen oxidation activity was detected in *Shewanella acanthi* and *Insolitispirillum peregrinum*. *Burkholderia plantarii* exhibited low but reproducible hydrogen oxidation activity despite the absence of a clear hydrogenase candidate in the annotation‐based search applied here. This observation prompted further screening of *Paraburkholderia mimosarum*, *P. xenovorans*, *P. kururiensis*, *P. phenoliruptrix*, and *P. phymatum*, revealing hydrogen oxidation activity across these related strains. Among the initially investigated nonmodel organisms, *I. peregrinum* exhibited the highest hydrogen oxidation activity and showed prolonged activity, partial oxygen tolerance, and substantial activity at elevated temperatures. Hydrogen production was consistently lower than hydrogen oxidation under the applied assay conditions. Overall, the workflow provides a practical approach for comparative screening of hydrogen‐converting activity in native bacterial fractions and can support the selection of candidate systems for further biochemical characterization.

## Graphical abstract

A standardized gas chromatography–based workflow enables direct functional screening of hydrogenase activity in crude membrane and lysate fractions from native bacteria. Without purification or heterologous expression, hydrogen oxidation is detected across phylogenetically diverse organisms, highlighting the value of activity‐based screening for functional enzyme discovery.

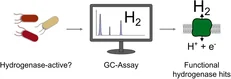

## 1 Introduction

The global transition toward a sustainable energy economy necessitates the development of efficient, carbon‐neutral energy carriers [1]. Molecular hydrogen (H_2_) is of particular interest due to its high energy density and clean conversion into water [2]. The controlled oxidation of H_2_ is relevant for applications such as fuel cells, where its chemical energy is converted into electricity. In this context, biocatalytic H_2_ oxidation offers an attractive alternative to conventional noble‐metal‐based catalysis as highly efficient conversion can be achieved under mild reaction conditions while reducing the dependence on expensive metals such as platinum [3, 4]. Central to this approach are hydrogenases, metalloenzymes capable of catalyzing the reversible oxidation of molecular hydrogen into protons and electrons. Among these, [NiFe]‐hydrogenases are particularly notable for their preference for H_2_ oxidation and for their O_2_‐tolerance [5].

The rapid expansion of genomic databases has led to the identification of a vast number of putative hydrogenase sequences across phylogenetically diverse microorganisms [6]. Large‐scale bioinformatic resources such as HydDB have further facilitated the classification and annotation of hydrogenases based on sequence similarity [7]. However, sequence‐based annotation alone does not guarantee functional activity [8], and a substantial gap remains between predicted hydrogenase genes and experimentally validated enzymes. This gap is particularly pronounced because hydrogenase activity depends not only on the structural integrity of the enzyme but also on correct cofactor maturation, the presence of accessory proteins required for metal insertion, and, in many cases, stringent control of oxygen exposure [9].

Heterologous expression provides an established route for the production and functional characterization of hydrogenases but can be experimentally demanding because activity often depends on correct cofactor assembly and the presence of specific maturation and accessory proteins. Common hosts such as *Escherichia coli* may therefore require co‐expression of the hydrogenase structural genes together with the corresponding maturation machinery to obtain active recombinant enzymes [6, 10, 11, 12]. Functional heterologous expression and purification have been successfully demonstrated for several hydrogenases, although the required systems can involve the coordinated expression of numerous genes and considerable experimental optimization [13, 14]. Direct activity screening in native cellular fractions provides a complementary approach that allows the hydrogen‐converting activity to be assessed without first establishing a heterologous expression system.

To address this challenge, a range of experimental approaches has been developed to assess hydrogenase activity. Photometric assays based on artificial electron acceptors such as benzyl viologen (BV) or methyl viologen (MV) are widely used due to their simplicity and compatibility with high‐throughput screening formats [15, 16, 17]. However, these assays provide only an indirect readout of hydrogenase activity and are therefore inherently limited in specificity [18]. In whole‐cell or crude extract formats, the measured signal can be influenced not only by enzymatic activity itself but also by enzyme maturation state, metal availability, growth phase, and the accessibility of the mediator to the enzyme. Moreover, other redox‐active enzymes can reduce viologens, leading to false‐positive signals [19, 20, 21].

Electrochemical approaches provide a more direct mechanistic readout by coupling hydrogenases to electrode surfaces and monitoring catalytic currents under controlled potentials [22, 23]. While highly informative, these methods typically require enzyme immobilization on tailored electrode materials and often involve specific surface functionalization strategies. As a result, the catalytic performance can be strongly influenced by the electrode interface, and experimental setups are not easily standardized. This limits comparability across studies and complicates routine measurements of crude or heterogeneous samples.

Taken together, currently available assay systems differ substantially in terms of specificity, experimental complexity, and compatibility with native biological samples. Photometric methods offer high throughput but rely on indirect readouts, whereas electrochemical approaches provide mechanistic insight at the expense of experimental accessibility and standardization. Gas chromatography (GC) represents a complementary strategy that enables direct quantification of molecular hydrogen while remaining compatible with complex biological samples.

Here, we establish a standardized GC‐based workflow for screening hydrogen‐converting activity in crude membrane and lysate fractions derived from native bacterial samples (Figure 1). The workflow directly quantifies H_2_ consumption or production under defined assay conditions without requiring enzyme purification or the prior establishment of a heterologous expression system. It was applied to a panel of bacterial strains representing different hydrogenase backgrounds and benchmarked using well‐characterized reference organisms. Importantly, the workflow is intended as a functional screening approach and does not by itself identify the underlying hydrogenase genes, determine the enzyme expression or maturation status, or establish the molecular origin of the observed activity. Instead, it provides a standardized means to identify native bacterial fractions exhibiting hydrogen‐converting activity and thereby select candidate systems for subsequent biochemical and molecular characterization.

**FIGURE 1:**
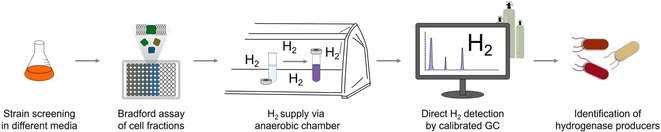
Schematic overview of the GC‐based workflow for hydrogenase activity assessment in native bacterial fractions. Following cultivation, membrane fractions or cell lysates were prepared and protein concentrations were normalized using the Bradford assay. Samples were transferred into an anaerobic chamber and combined with an artificial electron acceptor prior to sealing in gas‐tight vials under a defined H_2_‐containing headspace. The electron acceptor served to couple hydrogen oxidation to a continuous electron sink, while catalytic activity was quantified independently by direct gas chromatographic measurement of H_2_ consumption in the headspace. The workflow was applied to phylogenetically diverse bacterial strains representing both annotation‐supported and exploratory cases.

## 2 Results and Discussion

### 2.1 Strain Selection and Growth Analysis

To systematically evaluate the applicability of the GC‐based workflow across different biological contexts, a set of bacterial strains was selected comprising both annotation‐supported hydrogenase systems and an exploratory case lacking a clear sequence‐based prediction (Table 1). The *E. coli* strains MC4100 and FTD147 were included as well‐characterized reference systems. MC4100 possesses functional hydrogenases, whereas FTD147 lacks hydrogenases 1–3 and served as a low‐background control under the applied conditions. Annotation‐based inspection of the genomes of *Insolitispirillum peregrinum* and *Shewanella acanthi* identified putative [NiFe]‐hydrogenases (Table 1). In *I. peregrinum*, two distinct hydrogenase candidates were detected, corresponding to a membrane‐associated and a soluble variant, whereas *S. acanthi* encoded a single putative membrane‐associated hydrogenase. In contrast, no clearly annotated hydrogenase candidate was identified in *Burkholderia plantarii*. Nevertheless, *B. plantarii* was included as an exploratory strain based on the indications from independent bioinformatic analyses. Recent advances in protein embedding‐based targeted function discovery illustrate the potential of such approaches to identify hydrogenase candidates that may escape conventional sequence‐ and profile‐based annotation methods [27].

**TABLE 1:** Strains used in this study, cultivation conditions, and hydrogenase background.

Cultivation conditions						
**Organism**	**Hydrogenase type(s)**	**Growth tempera‐ture, °C**	**Cultivation time, h**	**Medium**	**Condition**	**Source**
*Burkholderia plantarii*	No clearly annotated hydrogenase candidate	30	72	LB	Atmospheric	DSMZ 9509
*Paraburkholderia mimosarum*	[NiFe]	28	72	DSMZ 830	Atmospheric	DSMZ 21841
*Paraburkholderia phenoliruptrix*	[NiFe]	28	72	DSMZ 1	Atmospheric	DSMZ 17773
*Paraburkholderia phymatum*	[NiFe]	28	72	LB	Atmospheric	DSMZ 17167
*Paraburkholderia kururiensis*	[NiFe]	28	72	DSMZ 545	Atmospheric	DSMZ 13646
*Paraburkholderia xenovorans*	[NiFe]	28	72	DSMZ 220	Atmospheric	DSMZ 17367
*Escherichia coli* MC4100 (all hydrogenases are functional)	[NiFe]	37	27	TB	Anaerobic	Sawers & Sargent [24, 25]
*Escherichia coli* FTD147 (MC4100 Δ*hyaB* Δ*hybC* Δ*hycE*)	[NiFe]	37	27	TB	Anaerobic	Sawers & Sargent [25, 26]
*Clostridium pasteurianum*	[NiFe] and [FeFe]	37	27	DSMZ 54b	Anaerobic	DSMZ 525
*Bacillus subtilis*	No native hydrogenase	30	27	LB	Atmospheric	Boca scientific host strain 1012 wt (PBS020)
*Nitratidesulfo‐vibrio vulgaris* Hildenborough	[NiFe] and [FeFe]	37	170	DSMZ 63	Anaerobic	DSMZ 644
*Cupriavidus necator*	[NiFe]	30	170	H16	Atmospheric	DSM 428
*Insolitispirillum peregrinum*	[NiFe]	30	27	DSMZ 37	Atmospheric	DSMZ 1839
*Insolitispirillum peregrinum* subsp. *integrum*	[NiFe]	30	27	DSMZ 37	Atmospheric	DSMZ 11589
*Shewanella acanthi*	[NiFe]	30	27	LB	Atmospheric	KCTC 82650

**[NiFe]‐hydrogenase candidates analyzed in this study**			
**Host organism**	**Reference**	**Localization**	**Sequence identity to *E. coli* hydrogenases**
*Insolitispirillum peregrinum*	WP_076401456.1	Membrane	~65%
*Insolitispirillum peregrinum*	WP_076400179.1	Cytoplasm	~35%
*Shewanella acanthi*	WP_220052144.1	Membrane	~45%
*Burkholderia plantarii*	N.a.	N.a.	n.a.
*Paraburkholderia mimosarum*	WP_028215130.1	Membrane	~61%
*Paraburkholderia phenoliruptrix*	WP_015004151.1	Membrane	~72%
*Paraburkholderia phymatum*	WP_012406417.1	Membrane	~79%
*Paraburkholderia kururiensis*	WP_017774733.1	Cytoplasm	~37%
*Paraburkholderia xenovorans*	WP_011493687.1	Cytoplasm	~46%

Following strain selection, cultivation conditions were systematically evaluated to establish reproducible growth protocols enabling biomass generation for hydrogenase activity assays. *S. acanthi* is known to grow in conventional LB medium [28], which yielded sufficient biomass under the tested conditions.

Two closely related strains, *I. peregrinum* and *I. peregrinum* subsp. *integrum* were initially evaluated to identify suitable cultivation conditions and determine which strain provided sufficient biomass for subsequent hydrogenase activity screening. Phenotypic differences between closely related bacterial strains or subspecies, such as *Parageobacillus thermoglucosidasius* [29] and *Alcaligenes eutrophus* [30], have been reported previously, supporting the experimental evaluation of both strains rather than assuming comparable cultivation behavior. As no growth was observed in LB or TB medium, four recommended DSMZ media (7, 37, 790, and 830) were evaluated. *I. peregrinum* showed superior growth under the tested conditions and was therefore selected for the subsequent hydrogenase activity assays.

Due to substantially reduced growth under anaerobic conditions, both *Insolitispirillum* strains were cultivated under atmospheric conditions. All experiments were performed at 30 °C as preliminary tests at 40 °C did not support growth. Cultures were inoculated to a standardized starting optical density (OD_600_ = 0.05), and growth performance was assessed by endpoint biomass determination after 24 h and by high‐resolution growth monitoring over 50 h (Figure S1).

Both *Insolitispirillum* strains reached the highest OD_600_ values in DSMZ medium 37 and showed moderate growth in media 790 and 830, while only minimal biomass formation was observed in medium 7. Media 37 and 790 contain elevated peptone concentrations (10 g L^−1^), suggesting a preference for complex nutrient sources, whereas media 7 and 830 provide only low glucose concentrations (≤1 g L^−1^), likely limiting biomass accumulation.

High‐resolution growth analysis provided additional insight into growth dynamics. Both strains exhibited negligible lag phases due to precultivation in the respective media. *I. peregrinum* displayed sustained exponential growth for approximately 15 h, followed by a stationary phase extending to approximately 40 h after inoculation. A slight increase in OD_600_ during late cultivation was observed, potentially associated with the formation of coccoid bodies under nutrient‐limited conditions [31]. In contrast, *I. peregrinum* subsp. *integrum* exhibited a shorter exponential phase of approximately 10 h and a decline in OD_600_ after approximately 30 h. Based on these results, DSMZ medium 37 and *I. peregrinum* were selected for all subsequent experiments.

A comparison of OD_600_ values between the two cultivation formats revealed a notable difference. The growth curves were recorded in 96‐well microplates, whereas the endpoint measurements were obtained from cultures grown in Erlenmeyer flasks. These cultivation formats differ in parameters such as aeration, gas–liquid transfer, and mixing efficiency, which can affect bacterial growth. The lower OD_600_ observed in the microplate cultivation may therefore result from differences in the cultivation conditions, including reduced aeration and mixing compared with flask cultivation.

### 2.2 Hydrogen Oxidation

Following the establishment of reproducible cultivation conditions, all strains yielded sufficient biomass for hydrogenase activity measurements. To validate the reliability and specificity of the GC‐based assay and to define suitable experimental conditions, membrane fractions of two well‐characterized *E. coli* reference strains were analyzed.

The strain MC4100 possesses functional hydrogenases, whereas the mutant strain FTD147 lacks hydrogenases 1–3 (Δ*hyaB*, Δ*hybC*, Δ*hycE*) and retains only the *hyd‐4* operon (*hyf*). Consistent with its genotype and the applied fermentative growth conditions [32, 33], FTD147 showed only minimal hydrogen oxidation under all tested conditions and was therefore used as a low‐background control (Figure S2). FTD147 nevertheless provides an important reference because it was derived directly from *E. coli* MC4100, thereby enabling a direct comparison with the hydrogenase‐positive parental strain. In addition, *Bacillus subtilis* was included as an independent negative control lacking annotated hydrogenases. *B. subtilis* showed negligible H_2_ oxidation under standard assay conditions and only a low background signal at increased H_2_ availability.

In the assay, molecular hydrogen was oxidized to protons and electrons, and the electrons were transferred to benzyl viologen (BV; 5 mM), which was used as the standard artificial electron acceptor. Hydrogen oxidation was quantified directly by GC as the decrease in headspace H_2_. Calibration with defined gas standards enabled reliable quantification over a range from approximately 4 µM to 5 mM H_2_ (Figure S3).

Compared to direct gas purging, the use of ambient hydrogen in an anaerobic chamber (~1000 µM H_2_) substantially simplified handling, enabled simultaneous incubation of multiple samples under uniform conditions, and prevented oxygen exposure. This setup provided a practical balance between analytical sensitivity and experimental throughput.

In addition, GC‐based analysis could be applied directly to crude membrane and lysate fractions without prior purification, thereby reducing limitations associated with restricted accessibility of externally supplied electron acceptors, such as BV, to hydrogenases in intact cells [21]. The use of cellular fractions may additionally reduce physiological constraints arising from differences in the growth state or cellular metabolism [34].

Sample preparation was performed under atmospheric conditions to simplify handling and broaden the applicability of the screening workflow without requiring anaerobic equipment during cell disruption and fractionation. However, this procedural choice may reduce or abolish the detectable activity of strongly O_2_‐sensitive hydrogenases and therefore represents a limitation when comparing hydrogenases with different O_2_ sensitivities. All prepared fractions were subsequently incubated under anaerobic conditions for 3.5 h prior to activity measurements; while this establishes anaerobic assay conditions, irreversible O_2_‐induced inactivation during sample preparation cannot be excluded. Accordingly, the workflow identifies hydrogenase activity retained after the standardized preparation procedure rather than the maximum intrinsic activity of the respective hydrogenase system.

Using MC4100 as a reference, reaction time and protein concentration were systematically evaluated (Figure 2A). Hydrogen consumption was detectable after 1 h, whereas 24 h of incubation yielded a substantially stronger signal and was therefore selected as the standard reaction time. This extended incubation was particularly advantageous for detecting weaker activities in nonmodel strains that might remain below the detection threshold at shorter incubation times. Protein concentrations were normalized using the Bradford assay, and decreasing protein input resulted in reduced hydrogen oxidation. Based on these results, a standard condition of 5 mg of protein mL^−1^ in a 5 mL reaction volume was selected for all subsequent experiments. Although normalization to total protein likely underestimates specific hydrogenase activities, it enabled rapid and standardized comparison across crude cellular fractions.

**FIGURE 2:**
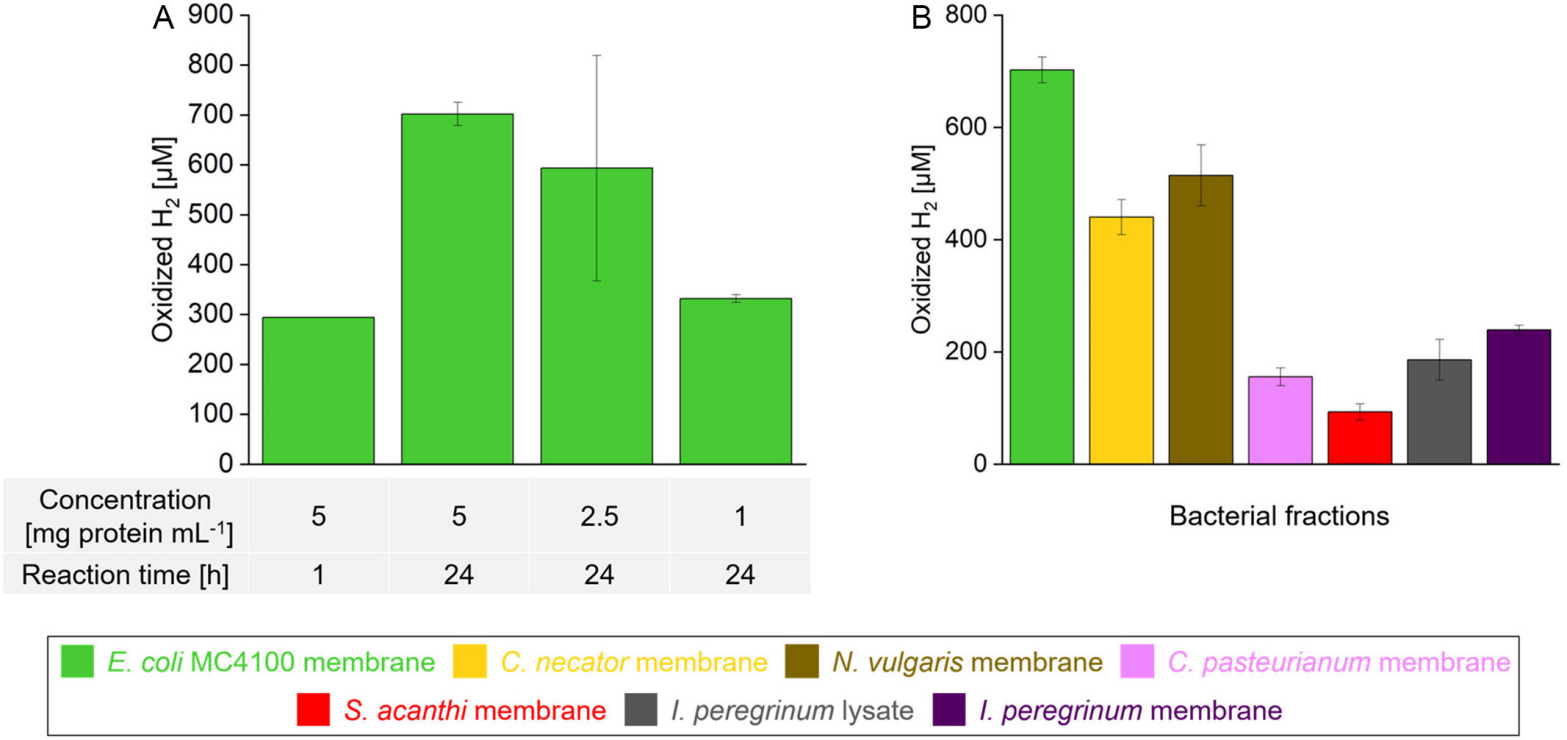
Assay optimization and comparative analysis of hydrogen oxidation. Assays were performed at 37 °C in sealed 20 mL vials containing a 5 mL reaction volume and 5 mM benzyl viologen (BV) as electron acceptor. Protein concentrations were determined prior to the assay using the Bradford method. (A) Optimization of incubation time and protein concentration using membrane fractions of the *E. coli* reference strain MC4100 under anaerobic chamber conditions (~1000 µM H_2_). Hydrogen oxidation was assessed after 1 and 24 h at 5 mg protein mL^−1^ and after 24 h at protein concentrations of 2.5 and 1 mg mL^−1^. (B) Comparative analysis of hydrogen oxidation in selected bacterial membrane and lysate fractions using a standardized protein concentration of 5 mg mL^−1^ and an incubation time of 24 h (~1000 µM initial H_2_). For direct comparison, the corresponding MC4100 measurement from panel (A) is included. Error bars represent the standard deviation of two independent experiments.

Following assay optimization, the workflow was applied to *S. acanthi* and *I. peregrinum* to evaluate its applicability across phylogenetically distinct organisms (Figure 2B). For *S. acanthi*, only the membrane fraction was analyzed, which is consistent with the presence of a single predicted membrane‐associated hydrogenase. In *I. peregrinum*, both soluble lysate and membrane fractions were examined, reflecting the presence of two distinct hydrogenase candidates (Table 1).

Comparative analysis revealed higher hydrogen oxidation activity in the membrane fraction of *I. peregrinum* than in the soluble lysate fraction, with both fractions exhibiting higher overall activity levels than those of *S. acanthi* (Figure 2B). Heat‐denatured control samples showed no detectable hydrogen oxidation (Figure S4), confirming that the observed signals originate from enzymatically active proteins.

Although the primary objective of this study was to investigate H_2_ oxidation by [NiFe]‐hydrogenases, *Clostridium pasteurianum* and *Nitratidesulfovibrio vulgaris* Hildenborough were additionally included. These strains are known to possess distinct types of hydrogenases [35, 36] and thus serve as reference organisms to assess the applicability of the workflow to strains harboring different hydrogenase types, including [NiFe]‐ and [FeFe]‐hydrogenases (Figure 2B). In addition, the model membrane‐bound hydrogenase of *Cupriavidus necator* was investigated to further validate our GC‐based screening approach. The strain was cultivated as previously described [37], and the membrane fraction showed comparable activity to *N. vulgaris* (Figure 2B).

The successful detection of hydrogenase activity in phylogenetically diverse organisms demonstrates the robustness of the GC‐based workflow and highlights its suitability for the comparative functional assessment of hydrogen‐converting enzymes in native cellular fractions.

### 2.3 Biochemical Characterization of the *I. peregrinum* Hydrogenase

As *I. peregrinum* exhibited the highest hydrogen oxidation activity, it was selected for further biochemical characterization. The suitability of different artificial electron acceptors was first evaluated (Figure 3A). In addition to BV, methylene blue and neutral red were tested as alternative mediators [38]. BV supported more than twofold higher hydrogen oxidation activity compared to the alternative electron acceptors, consistent with its more negative redox potential, which thermodynamically favors electron transfer from hydrogenases [39, 40].

**FIGURE 3:**
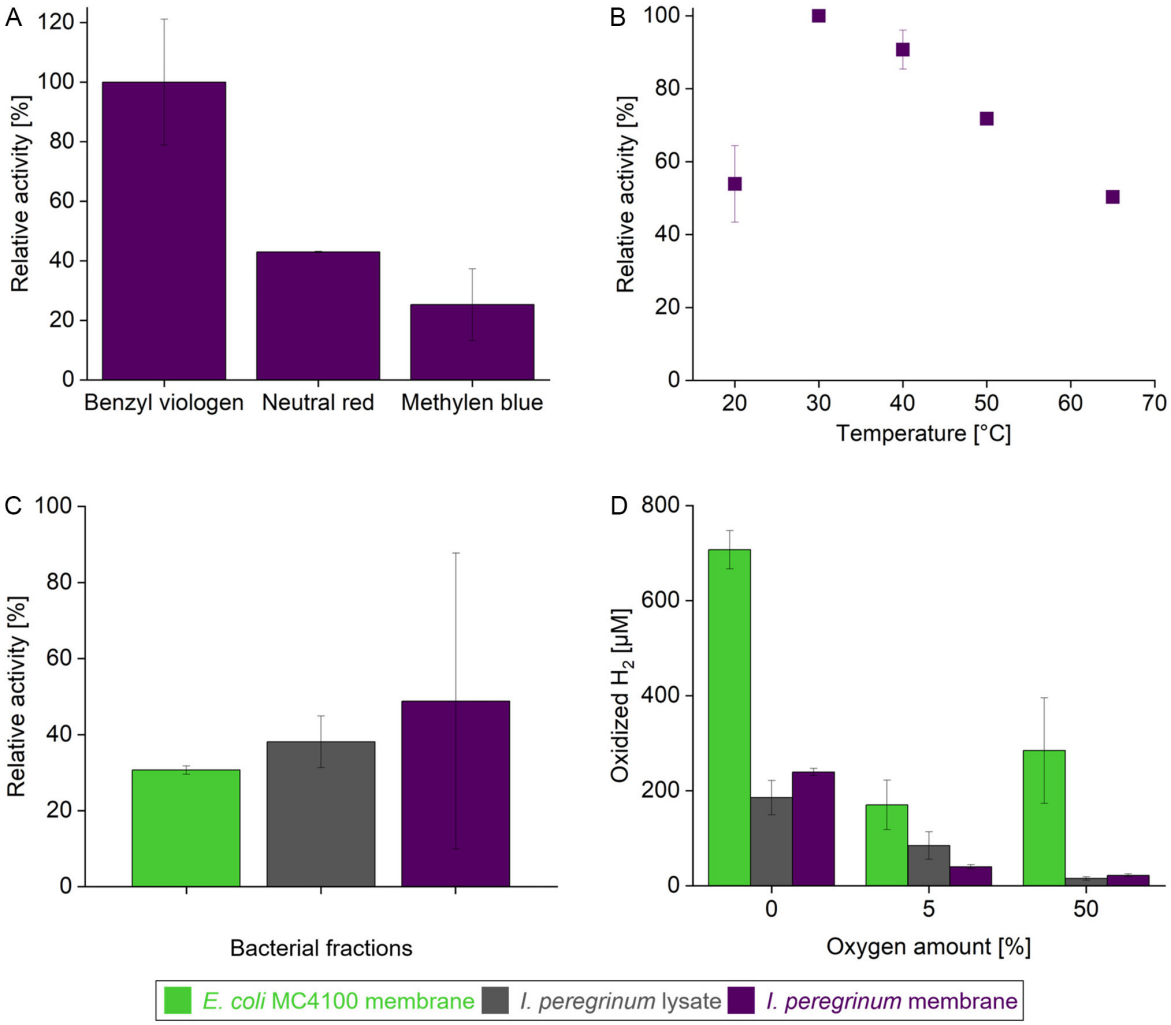
Biochemical characterization of hydrogenase activity and stability in *I. peregrinum*. (A) Effect of alternative electron acceptors on hydrogen oxidation activity. All electron acceptors were applied at 5 mM, and activity measured with benzyl viologen (BV) was set to 100%. (B) Temperature dependence of hydrogen oxidation activity. Reactions were incubated at the indicated temperatures for 24 h prior to GC analysis; activity at 30 °C was defined as 100%. (C) Long‐term stability. Membrane and soluble fractions of *I. peregrinum* and *E. coli* were stored anaerobically at room temperature for 3.5 h and 7 days prior to activity measurement. Activities measured after 3.5 h served as the reference and were set to 100%; the values shown after 7 days represent the remaining activity relative to this initial measurement. (D) Oxygen tolerance. Defined relative oxygen levels were generated by mixing oxygen‐saturated buffer with anaerobic buffer prior to sealing. The indicated values (0%, 5%, and 50%) refer to the fraction of oxygen‐saturated buffer in the reaction mixture and correspond to approximate dissolved oxygen concentrations of ~0, ~55−60, and ~550–600 µM, respectively. Data for 0% O_2_ correspond to standard assay conditions (5 mg protein mL^−1^, 24 h incubation under anaerobic chamber H_2_). Error bars represent the standard deviation of at least two independent experiments.

Temperature‐dependent measurements demonstrated that the maximal hydrogen oxidation activity coincided with the optimal growth temperature of *I. peregrinum* (Figure 3B). Notably, approximately 50% of the maximal activity remained detectable at 65 °C, indicating considerable thermal robustness. Such residual activity at elevated temperatures is consistent with previous observations that hydrogenases from mesophilic organisms can exhibit substantial thermostability [41]. The gradual decline in activity at higher temperatures is likely influenced not only by enzyme stability but also by reduced gas solubility, lowering substrate availability.

Subsequent experiments focused on parameters relevant for potential bioelectrochemical applications, in particular, long‐term stability and oxygen tolerance (Figure 3C,D) [42, 43]. To assess long‐term stability, membrane and soluble fractions were incubated under strictly anaerobic conditions and analyzed after 3.5 h and 7 days. For each sample, the activity measured after 3.5 h was defined as the reference (100%), and the activity measured after 7 days was expressed relative to this initial value. Thus, the values represent the fraction of the initial activity retained during storage rather than a comparison of absolute hydrogen oxidation activities between the strains. Both hydrogenase‐containing fractions from *I. peregrinum* retained approximately 40% of their initial activity after 7 days, whereas the *E. coli* sample retained approximately 30% (Figure 3C). Long‐term retention of hydrogenase activity is an important consideration for biotechnological applications, and high operational stability has been demonstrated in hydrogenase‐based catalytic systems [44]. The robustness of E. coli Hyd1 has likewise been demonstrated over extended reaction times and a broad temperature range [41]. Nevertheless, reported stabilities of [NiFe]‐hydrogenases vary considerably, ranging from nearly 100% activity after 45 days [45] to a 50% activity loss within 6 h [46].

In addition to long‐term stability, oxygen tolerance was evaluated as a second key parameter. Due to the explosion risk associated with direct mixing of H_2_ and O_2_, an indirect approach was employed, adapted from previously described methods [47, 48]. Oxygen‐saturated buffer was prepared separately, transferred gas‐tight into an anaerobic chamber, and mixed with anaerobic buffer to generate defined relative oxygen levels prior to sealing. The indicated values (0%, 5%, and 50%) refer to the fraction of oxygen‐saturated buffer in the reaction mixture rather than to the gas‐phase oxygen concentration. This corresponds to approximate dissolved oxygen concentrations of ~0 µM (0%), ~55–60 µM (5%), and ~550–600 µM (50%) at room temperature [49]. Across all tested conditions, *E. coli* exhibited the highest activity levels (Figure 3D), consistent with the known oxygen tolerance of its native hydrogenases [50]. Hydrogen oxidation activity of *I. peregrinum* remained detectable at elevated oxygen levels, indicating partial oxygen tolerance and suggesting potential adaptation to microaerobic conditions. However, as measurements were performed using crude cellular fractions rather than purified enzymes, contributions from auxiliary components cannot be excluded.

### 2.4 Detection of Hydrogen Oxidation Activity in (*Para*‐)*Burkholderia* Strains

In contrast to *S. acanthi* and *I. peregrinum*, *B. plantarii* represented an atypical case within the selected strain panel as no hydrogenase candidate was identified based on annotation (Table 1). *B. plantarii* is primarily known as a rice‐associated phytopathogen with a distinct repertoire of virulence and quorum‐sensing‐associated genes [51]. The strain was included as an exploratory case to assess whether the workflow can detect hydrogen‐converting activity in organisms lacking clear annotation‐based indications.

When the standardized assay established in Section 2.2 was initially applied, only low hydrogen oxidation activity was detected in membrane fractions. Since hydrogenase expression is often associated with nutrient limitation [52], the cultivation time was extended from 27 to 72 h. Under these conditions, measurable hydrogen consumption was observed under ambient H_2_ in the anaerobic chamber (Figure 4A). Although activity remained lower than that of *E. coli* MC4100 and *I. peregrinum*, it was reproducible and clearly distinguishable from the background signal of FTD147 and *B. subtilis* (Figure S2). The amount of oxidized H_2_ increased over time, indicating the presence of biologically relevant hydrogen‐oxidizing activity.

**FIGURE 4:**
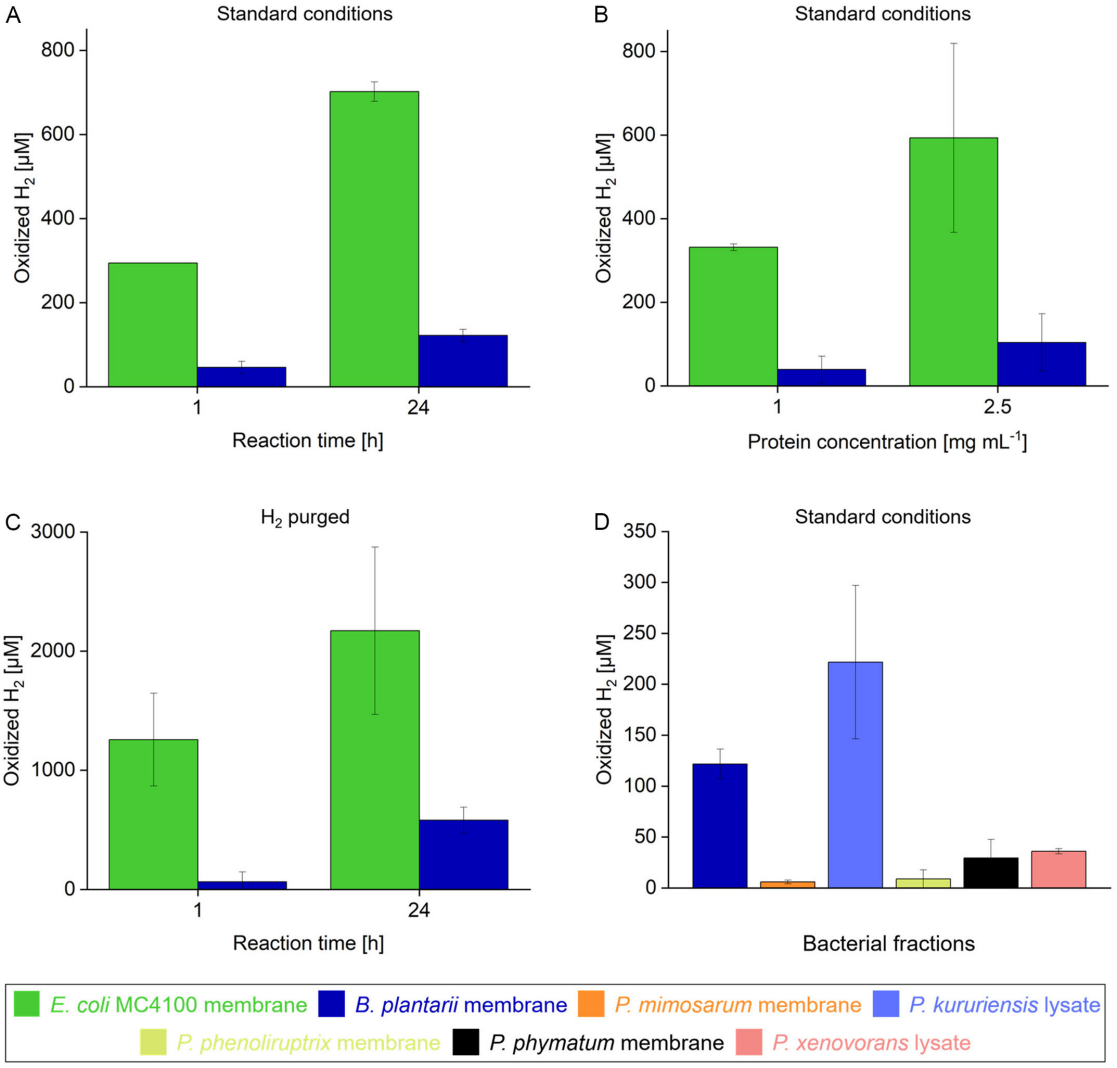
Hydrogen oxidation by *B. plantarii* and selected *Paraburkholderia* species. Assays were performed at 37 °C in 20 mL vials containing a 5 mL reaction volume and 5 mM benzyl viologen (BV) as electron acceptor. Protein concentrations were determined prior to the assay using the Bradford method. (A) Hydrogen oxidation under ambient headspace H_2_ (~1000 µM) after 1 and 24 h at 5 mg protein mL^−1^. (B) Hydrogen oxidation after 24 h at protein concentrations of 1 and 2.5 mg mL^−1^ under ambient headspace H_2_ (~1000 µM). (C) Hydrogen oxidation after 1 and 24 h at 5 mg protein mL^−1^ under increased H_2_ concentrations (~4500 µM) following gas purging. *E. coli* MC4100 was included as a positive control in panels A–C. (D) Hydrogen oxidation by *B. plantarii* and five *Paraburkholderia* species under ambient headspace H_2_ (~1000 µM) at 5 mg protein mL^−1^ after 24 h. *B. plantarii* was included as the reference strain. Error bars represent the standard deviation of two independent experiments.

To further assess this activity, the influence of the protein concentration was examined (Figure 4B). Hydrogen oxidation increased with increasing protein input, supporting the conclusion that the observed signal originated from a biological catalyst present in the cellular fraction rather than from analytical noise. However, the increase was not strictly proportional, indicating that the assay did not operate under ideal substrate‐saturating conditions across the investigated protein concentration range. Previous studies have shown that apparent hydrogenase activity can depend strongly on enzyme concentration and assay geometry, particularly in systems involving gas–liquid H_2_ transfer [53, 54]. In the present assay, additional factors such as H_2_ availability, gas–liquid mass transfer, accessibility of the hydrogenase within the cellular fraction, and electron transfer to BV may therefore influence the observed H_2_ conversion.

To evaluate the effect of substrate availability, experiments were repeated under H_2_‐purged conditions. Increasing the H_2_ concentration from ~1000 to ~4500 µM resulted in enhanced hydrogen oxidation by *B. plantarii* (Figure 4C). Under these conditions, *E. coli* MC4100 showed rapid hydrogen oxidation within 1 h (0.75 µmol h^−1^ mg^−1^ protein), consistent with the literature values obtained using photometric assays [55, 56]. FTD147 exhibited minimal activity (Figure S2), confirming its suitability as a control.

Together, these results demonstrate that the GC‐based assay is sufficiently sensitive to detect low levels of hydrogen oxidation activity in organisms lacking clear annotation‐based predictions. In the case of *B. plantarii*, such activity would likely remain undetected in strictly annotation‐driven workflows. While the genome of *B. plantarii* contains an annotated nitrogenase, and nitrogenases are capable of interacting with H_2_ [57], no physiological hydrogen oxidation rates comparable to those of hydrogenases have been reported. Accordingly, the observed activity should be interpreted conservatively as functional evidence for hydrogen oxidation rather than as a definitive assignment to a specific enzyme class.

This activity prompted us to investigate whether related members of the family Burkholderiaceae show comparable hydrogen oxidation activity. HydDB contains several Burkholderia‐lineage genomes with annotated [NiFe]‐hydrogenases. These strains are now assigned to the genus *Paraburkholderia* based on phylogenomic analyses separating predominantly environmental species from the mainly pathogenic lineages retained within *Burkholderia* [58]. *Paraburkholderia* species are metabolically versatile bacteria associated with soil and plants, and many are capable of beneficial plant interactions, including biological nitrogen fixation [59, 60]. Their occurrence in plant‐associated and nitrogen‐fixing systems makes hydrogenase activity particularly relevant as H_2_ metabolism may be linked to H_2_ production by nitrogenases [61].

Based on their hydrogenase annotations and their ecological relevance [62, 63], *P. mimosarum*, *P. xenovorans*, *P. kururiensis*, *P. phenoliruptrix*, and *P. phymatum* were selected for experimental characterization. Following acquisition from the DSMZ, the strains were cultivated under the recommended conditions to obtain sufficient biomass (Table 1) and subsequently analyzed using the standard hydrogenase activity screening (Figure 4D). According to the predicted hydrogenase localizations, membrane fractions were analyzed for potentially membrane‐bound hydrogenases, whereas the lysate was analyzed for soluble hydrogenases (Table 1). *P. kururiensis* showed the highest activity of all strains tested, exceeding that of *B. plantarii*, which served as the reference strain (Figure 4D).

For *P. phymatum*, previous studies have demonstrated the expression of the hydrogenase genes, providing evidence for the presence of an active hydrogenase system [64, 65]. Our results now provide direct experimental evidence that this hydrogenase is functionally active. A closer examination of the physiological conditions described in previous studies may help to identify cultivation conditions that further promote hydrogenase expression and activity and could thus increase the activity observed in this assay.

The low activities of *P. mimosarum* and *P. phenoliruptrix* may reflect suboptimal cultivation or induction conditions rather than the absence of functional hydrogenases. Hydrogenase expression and maturation depend on physiological conditions, oxygen availability, medium composition, and nickel availability [26, 66]. Future optimization should therefore evaluate these factors together with the potential role of the hydrogenases in recycling nitrogenase‐derived H_2_ during nitrogen fixation.

The detection of hydrogen oxidation activity across different (*Para*‐)*Burkholderia* strains illustrates the value of combining standardized functional assays with broader organism selection and demonstrates the applicability of the workflow to bacterial strains with different levels of prior hydrogenase annotation.

### 2.5 Hydrogen Production

Motivated by the reversible catalytic nature of hydrogenases [67] and to complement the hydrogen oxidation data, hydrogen production was evaluated across the investigated strains. Initial experiments focused on *B. plantarii* and *E. coli* as a reference system, followed by comparative analysis of the remaining organisms.

Hydrogen production was quantified using MV reduced by sodium dithionite as an electron donor (Figure 5A). For *E. coli*, production rates of 3.5 µmol h^−1^ mg protein^−1^ were observed, consistent with literature values ranging from 2.7 [68] to 6.6 [69] µmol h^−1^ mg protein^−1^. FTD147 did not show detectable hydrogen‐producing activity under the tested conditions (data not shown).

**FIGURE 5:**
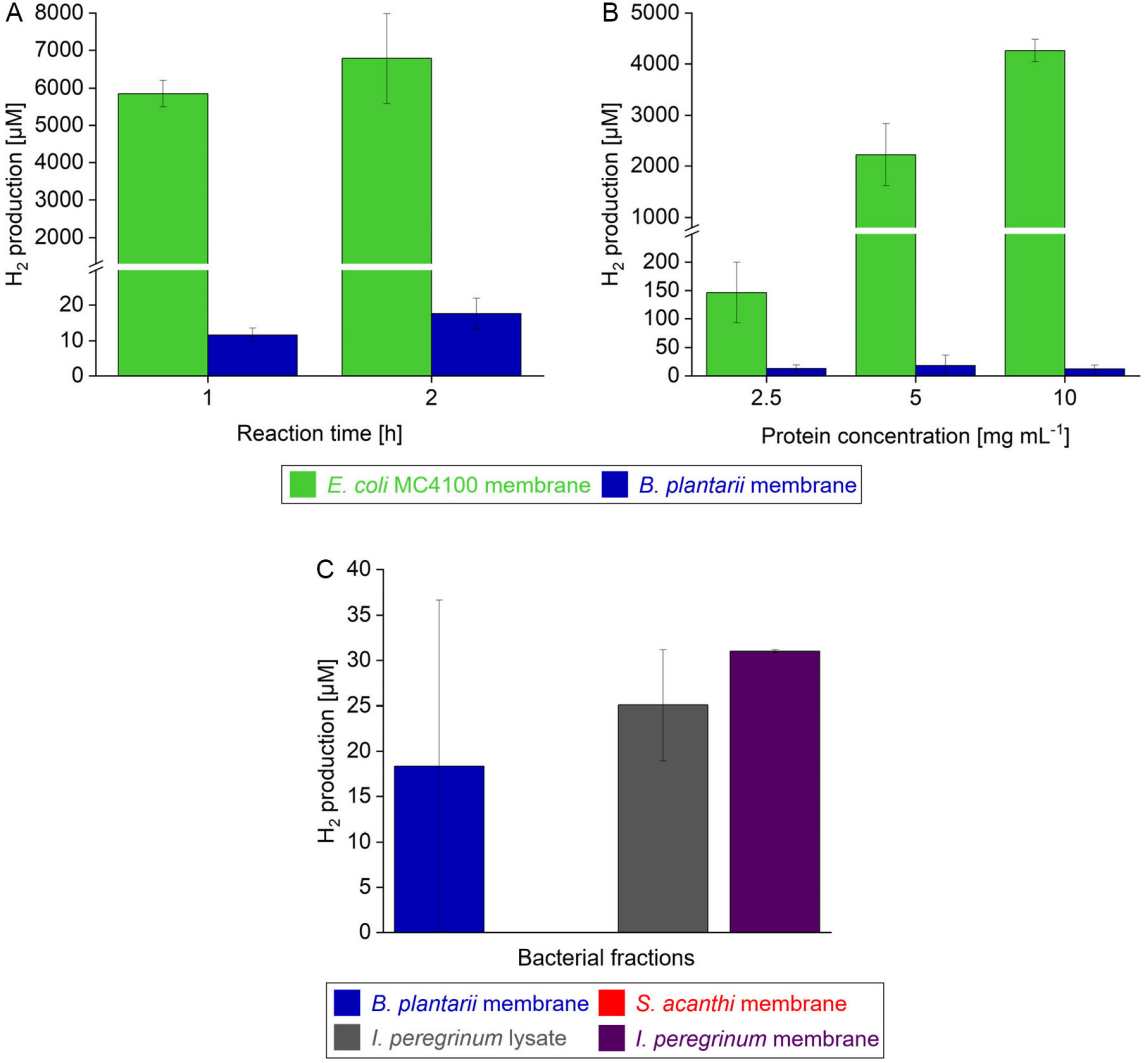
Assessment of hydrogen production activity. (A) Time‐dependent hydrogen production measured at 5 mg protein mL^−1^ after 1 and 2 h of incubation. (B) Protein‐concentration‐dependent hydrogen production after 24 h of incubation. (C) Comparative hydrogen production of *S. acanthi* membrane fractions and *I. peregrinum* membrane fractions and lysates after 24 h at 5 mg protein mL^−1^; the corresponding *B. plantarii* data from panel (B) are included for reference. *E. coli* MC4100 was included as a positive control in panels (A) and (B). All reactions were initiated by the addition of 10 mM MV and 100 mM sodium dithionite and incubated at 37 °C. Error bars represent the standard deviation of two independent experiments.

To characterize H_2_ production at early time points, initial measurements were conducted after 1 and 2 h of incubation (Figure 5A). Substantial H_2_ production by the *E. coli* reference sample was already observed within this period. These measurements were intended to assess early H_2_ production rather than to determine the time point of maximum production. For comparative screening across different protein concentrations and bacterial samples, a standardized 24 h endpoint was subsequently used (Figure 5B,C), providing consistent assay conditions for comparison across the investigated samples.

A clear dependence of hydrogen production on the protein concentration was observed for *E. coli*, confirming the reliability of the assay across varying enzyme levels. In contrast, production rates in *B. plantarii* showed only a weak dependence on the protein concentration, consistent with its overall lower catalytic activity. Based on these observations, subsequent assays were performed at a standardized protein concentration of 5 mg mL^−1^, consistent with the oxidation experiments.

Comparison with hydrogen oxidation activities revealed consistently lower production rates across all investigated strains. In particular, hydrogen production by *B. plantarii* (7 nmol h^−1^ mg protein^−1^; Figure 5A) remained substantially below its corresponding oxidation rate (~39 nmol h^−1^ mg protein^−1^; Figure 4C). A similar trend was observed for *I. peregrinum*, whereas *S. acanthi* exhibited activity exclu‐sively in the direction of hydrogen oxidation (Figures 5C and 2B).

These results indicate a pronounced functional bias toward hydrogen oxidation among the investigated systems. This observation highlights the importance of assessing both reaction directions in functional screening workflows as analysis restricted to a single reaction mode may lead to incomplete characterization of hydrogen‐converting activity.

## 3 Conclusion

The present study establishes a standardized GC‐based workflow for the direct assessment of hydrogenase activity in native cellular fractions, enabling functional validation across phylogenetically diverse organisms without the need for enzyme purification or heterologous expression. By applying this approach to a defined panel of bacterial strains, hydrogen oxidation activity was detected in *S. acanthi*, *I. peregrinum*, and *B. plantarii*, demonstrating that functionally relevant hydrogen conversion can be identified even in cases where sequence‐based evidence is limited or ambiguous.

Among the investigated strains, *I. peregrinum* exhibited the highest hydrogen oxidation activity and showed favorable operational properties, including prolonged activity, partial oxygen tolerance, and substantial activity at elevated temperatures. These characteristics highlight its potential as a source of hydrogen‐converting biocatalysts. At the same time, *E. coli* consistently outperformed all newly analyzed organisms and served as an important benchmark for assay validation and performance comparison.

The ability to detect hydrogen‐converting activity directly in crude membrane and lysate fractions underscores the sensitivity and robustness of the presented workflow. In particular, the detection of low but reproducible activity in *B. plantarii* illustrates the value of complementing annotation‐based approaches with systematic functional screening.

Taken together, this work demonstrates that standardized, fraction‐based activity assays provide a powerful strategy for functional validation of hydrogen oxidation. Expanding this approach to a broader range of organisms and cultivation conditions is expected to further extend the accessible diversity of hydrogen‐converting systems.

In addition, the workflow provides a basis for targeted assessment of functional properties relevant to bioelectrochemical applications, including O_2_ robustness [5, 27, 42, 43]. For example, comparing hydrogenase activity following controlled aerobic and strictly anaerobic sample preparation could enable the identification of candidates that retain activity after defined O_2_ exposure. Such comparative screening would complement bioinformatic approaches for identifying potentially interesting hydrogenase candidates and provide a rational basis for selecting enzymes for subsequent detailed biochemical characterization and biotechnological application.

## 4 Experimental Section

### 4.1 Selection of Strains and Hydrogenase Systems

A set of bacterial strains was selected to enable experimental validation of hydrogenase activity across both annotation‐supported and exploratory cases.

*E. coli* strains MC4100 and FTD147 were included as well‐characterized reference systems. MC4100 possesses functional hydrogenases, whereas FTD147 (MC4100 Δ*hyaB* Δ*hybC* Δ*hycE*) lacks hydrogenases 1–3 and served as a low‐background control under the applied conditions [24, 25, 26].

*S. acanthi* (taxon ID: 2864212) and *I. peregrinum* (taxon ID: 80 876) were selected as nonmodel organisms with annotated hydrogenase‐related genes identified by querying their respective NCBI genome entries using the keyword “hydrogenase”. In *I. peregrinum*, two distinct [NiFe]‐hydrogenase candidates were identified, whereas *S. acanthi* encoded a single putative membrane‐associated hydrogenase.

In contrast, no clearly annotated hydrogenase candidate was detected in the genome of *B. plantarii* (taxon ID: 41899) based on this approach. Nevertheless, *B. plantarii* was included as an exploratory case following indications from independent bioinformatic analyses suggesting that this strain might represent an interesting candidate for functional hydrogenase screening [27].

### 4.2 Bacterial Growth

For each strain, 100 mL of preculture was prepared in the respective growth medium and incubated overnight at 180 rpm under atmospheric conditions. On the following day, 40 mL of the preculture was used to inoculate 2 L of fresh medium. All strains used in this study and their corresponding cultivation conditions for hydrogenase production are summarized in Table 1.

LB and TB media were obtained commercially. The cultivation of *C. necator* [37] and *N. vulgaris* Hildenborough [70] was performed as previously described. The compositions of DSMZ (German Collection of Microorganisms and Cell Cultures) media are as follows:

DSMZ medium 37: 10 g L^−1^ peptone, 1 g L^−1^ succinic acid, 1 g L^−1^ (NH_4_)_2_SO_4_, 1 g L^−1^ MgSO_4_ × H_2_O, 0.002 g L^−1^ FeCl_3_ × 6 H_2_O, and 0.002 g L^−1^ MnSO_4_ × H_2_O; pH 6.8.

DSMZ medium 830: 0.5 g L^−1^ peptone, 0.5 g L^−1^ yeast extract, 0.5 g L^−1^ casamino acids, 0.5 g L^−1^ glucose, 0.5 g L^−1^ starch, 0.3 g L^−1^ Na‐pyruvate, 0.3 g L^−1^ K_2_HPO_4_, and 0.05 g L^−1^ MgSO_4_ × 7 H_2_O; pH 7.2.

DSMZ medium 7: 1 g L^−1^ peptone, 1 g L^−1^ glucose, and 1 g L^−1^ yeast extract; pH 7.0.

DSMZ medium 790: 10 g L^−1^ peptone, 1 g L^−1^ yeast extract, 2 g L^−1^ MgSO_4_ × 7 H_2_O, and 2 g L^−1^ (NH_4_)_2_SO_4_; pH 7.0.

DSMZ medium 1: 5 g L^−1^ peptone and 3 g L^−1^ meat extract; pH 7.

DSMZ medium 545: 2.5 g L^−1^ K_2_HPO_4_, 5 g L^−1^ NaCl, 2.5 g L^−1^ glucose, 3 g L^−1^ soy peptone, 17 g L^−1^ casein peptone; pH 7.3.

DSMZ medium 220: 5 g L^−1^ NaCl, 5 g L^−1^ peptone from soymeal, and 15 g L^−1^ peptone from casein; pH 7.3.

DSMZ medium 54b: 20 g L^−1^ glucose, 10 g L^−1^ yeast extract, and 20 g L^−1^ CaCO_3_; pH not adjusted.

After cultivation, cells were harvested under atmospheric conditions by centrifugation (10,000 × *g*, 10 min), resuspended in 100 mM potassium phosphate buffer (KPi; K_2_HPO_4_/KH_2_PO_4_, pH 8.0), and stored at −80 °C until further use.

For high‐resolution growth curve analysis, cultures were grown overnight in the respective medium at 180 rpm. Cultures were then diluted to an OD_600_ of 0.05, and 150 µL aliquots were transferred into a 96‐well plate. Measurements were performed at 30 °C using a Synergy H1 plate reader (BioTek Instruments, Germany).

### 4.3 Sample Preparation and Cell Fractionation

Sample preparation was performed under atmospheric conditions. A total of 10 mL of thawed cell suspension was treated with approximately 1 mg each of DNase and lysozyme and incubated for 30 min at room temperature on a rotary mixer. Cells were lysed by ultrasonication on ice using a Bandelin Sonopuls device (308 µm amplitude, ~50% power) in three cycles of 30 s, each followed by 30 s of cooling on ice.

The resulting suspension was centrifuged at 45,000 × *g* for 60 min at 4 °C to separate the membrane fraction (pellet including cell debris) from the soluble lysate fraction (supernatant). The membrane fraction was resuspended in 4 mL of 100 mM KPi buffer.

Protein concentrations of membrane and lysate fractions were determined using a Bradford assay, as described in Section 4.4, to enable analysis of hydrogenase activity in native cellular fractions.

### 4.4 Protein Quantification

Protein concentrations in membrane and lysate fractions were determined using a commercial Bradford assay [71] (Bio‐Rad) according to the manufacturer’s instructions. Bovine serum albumin (BSA) was used to generate a calibration curve for each experiment.

### 4.5 Hydrogen Oxidation Assay

Hydrogen oxidation assays were performed using crude membrane and lysate fractions prepared as described in Section 4.3. Samples were transferred into an anaerobic chamber (Figure S5A; 0 ppm O_2_, approximately 1000 µM H_2_, and the rest N_2_) and pre‐incubated for 210 min. This condition was defined as the standard method as molecular hydrogen was supplied directly from the chamber atmosphere without the need for additional gas handling.

Reactions were set up in 20 mL GC vials (Figure S5B; Agilent) containing 5 mL of 100 mM Britton–Robinson buffer (pH 6.0) [72]. Protein concentrations were adjusted to final concentrations of 1, 2.5, or 5 mg mL^−1^. Reactions were initiated by the addition of an electron acceptor dissolved in ddH_2_O. BV was used as the standard electron acceptor unless stated otherwise; neutral red and methylene blue were tested as alternatives. Electron acceptors were applied at a final concentration of 5 mM.

Reaction vials were sealed gas‐tight inside the anaerobic chamber and subsequently incubated at 37 °C and 180 rpm outside the chamber until analysis (see Section 4.7). Control samples containing buffer and an electron acceptor but no protein were prepared and handled identically.

For experiments with increased hydrogen availability, samples were flushed with a gas mixture containing approximately 4500 µM H_2_ (rest argon) for 120 s prior to sealing.

For oxygen tolerance experiments, buffer was pre‐saturated with O_2_, transferred into the anaerobic chamber, and mixed with anaerobic buffer to obtain defined oxygen concentrations prior to sealing.

### 4.6 Hydrogen Production Assay

Hydrogen production assays were performed using a protocol based on a method previously described [73]. Crude membrane and lysate fractions were prepared, as described in Section 4.3. Prior to the assay, samples were degassed by flushing with pure argon for at least 120 s to remove residual hydrogen and ensure anoxic conditions.

Reactions were set up in 20 mL GC vials containing 5 mL of 100 mM Britton–Robinson buffer (pH 6.0). Protein concentrations were adjusted as described for hydrogen oxidation assays (Section 4.4).

Reactions were initiated by the addition of methyl viologen (MV; 10 mM) and sodium dithionite (100 mM), supplied as 1000 mM stock solutions prepared in water. These concentrations have been commonly used in previous studies [74, 75]. For a final reaction volume of 5 mL, 50 µL of the MV stock solution and 500 µL of the sodium dithionite stock solution were added.

Reaction vials were sealed gas‐tight and incubated at 37 °C and 180 rpm until analysis (see Section 4.7). Control samples lacking protein were prepared and handled identically.

### 4.7 Hydrogen Quantification

Hydrogen was quantified in the headspace using an Agilent 8860 gas chromatograph equipped with a molecular sieve 5A column (mesh 80/100, 1.5 m × 1/16″ × 1 mm). Argon was used as the carrier gas at a flow rate of 24 mL min^−1^, with nitrogen as the makeup gas at 1 mL min^−1^. The oven and detector temperatures were set to 50 and 220 °C, respectively.

Hydrogen concentrations were determined quantitatively using a calibration curve generated with defined H_2_ concentrations in argon (Figure S3). Chromatogram peak areas (arbitrary units, AU) were converted to H_2_ concentrations using the linear regression obtained from the calibration data according to the following equation: H_2_ (µM) = [(peak area × 13.70725) + 54.824]/22.43, where 13.70725 and 54.824 represent the slope and intercept of the linear calibration, respectively, and 22.43 corresponds to the molar gas volume under standard conditions (22.43 L mol^−1^).

For hydrogen oxidation assays, the H_2_ concentration measured in each sample was compared with the corresponding control lacking the protein. The amount of oxidized H_2_ was calculated as the difference between the H_2_ concentration in the protein‐free control and that remaining in the sample after incubation [oxidized H_2_ = H_2_(control) − H_2_(sample)]. Thus, “oxidized H_2_” refers to the decrease in H_2_ relative to the corresponding protein‐free control and not to the residual H_2_ concentration in the vial. Apparent negative values after correction were set to zero. No corresponding reference correction was applied to the hydrogen production experiments.

To calculate the absolute amount of H_2_, a headspace volume of 15 mL was assumed for all reactions. Hydrogenase activity was calculated from the amount of H_2_ consumed or produced, the reaction time, and the protein amount used in the assay and was reported as µmol H_2_ h^−1^ mg protein^−1^.

Relative activity was calculated by setting the amount of oxidized H_2_ under the respective reference condition to 100% and expressing all other measurements relative to this value according to the following equation: relative activity (%) = [oxidized H_2_(sample)/oxidized H_2_(reference)] × 100. The respective reference condition is specified in the corresponding figure caption.

## Funding

This study was supported by the Helmholtz Association program “Materials Systems Engineering” under the topic “Adaptive and Bioinstructive Materials Systems” (Funding code: 43.33.11) and KIT EXU project “DigiteLiSE”.

## Conflicts of Interest

The authors declare no conflicts of interest.

## Supporting information

Supplementary Material

## Data Availability

The data that support the findings of this study are available from the corresponding author upon reasonable request.
