# Supplementary material for: A Gas Chromatography‐Based Workflow Leads to the Detection of Hydrogenase Activity in Native Bacteria
Source: Chembiochem. 2026 Sep 30;27(19):e70545. doi: 10.1002/cbic.70545 (PMC13626601; doi:10.1002/cbic.70545)
Supplement: Supplementary file 1 [file CBIC-27-e70545-s001.pdf]

Supporting Information

A Gas Chromatography-Based Workflow Leads to the Detection of Hydrogenase Activity in Native Bacteria

Marius Stoeckle [a], Kersten S. Rabe [a] and Christof M. Niemeyer [a]

a) Institute for Biological Interfaces 1 (IBG1), Karlsruhe Institute of Technology (KIT), D-76344 Eggenstein-Leopoldshafen, Germany

Table of contents

Supplementary Figures

Figure S1: Growth characteristics of *I. peregrinum* and *I. peregrinum* subsp. *integrum*

Figure S2: Hydrogen oxidation by *E. coli* FTD147 and *Bacillus subtilis* control strains

Figure S3: Calibration of H_2 quantification by gas chromatography

Figure S4: Effect of heat denaturation on hydrogen oxidation activity in *S. acanthi* and *I. peregrinum*

Figure S5: Experimental setup for hydrogen oxidation assays

Supplementary Figures

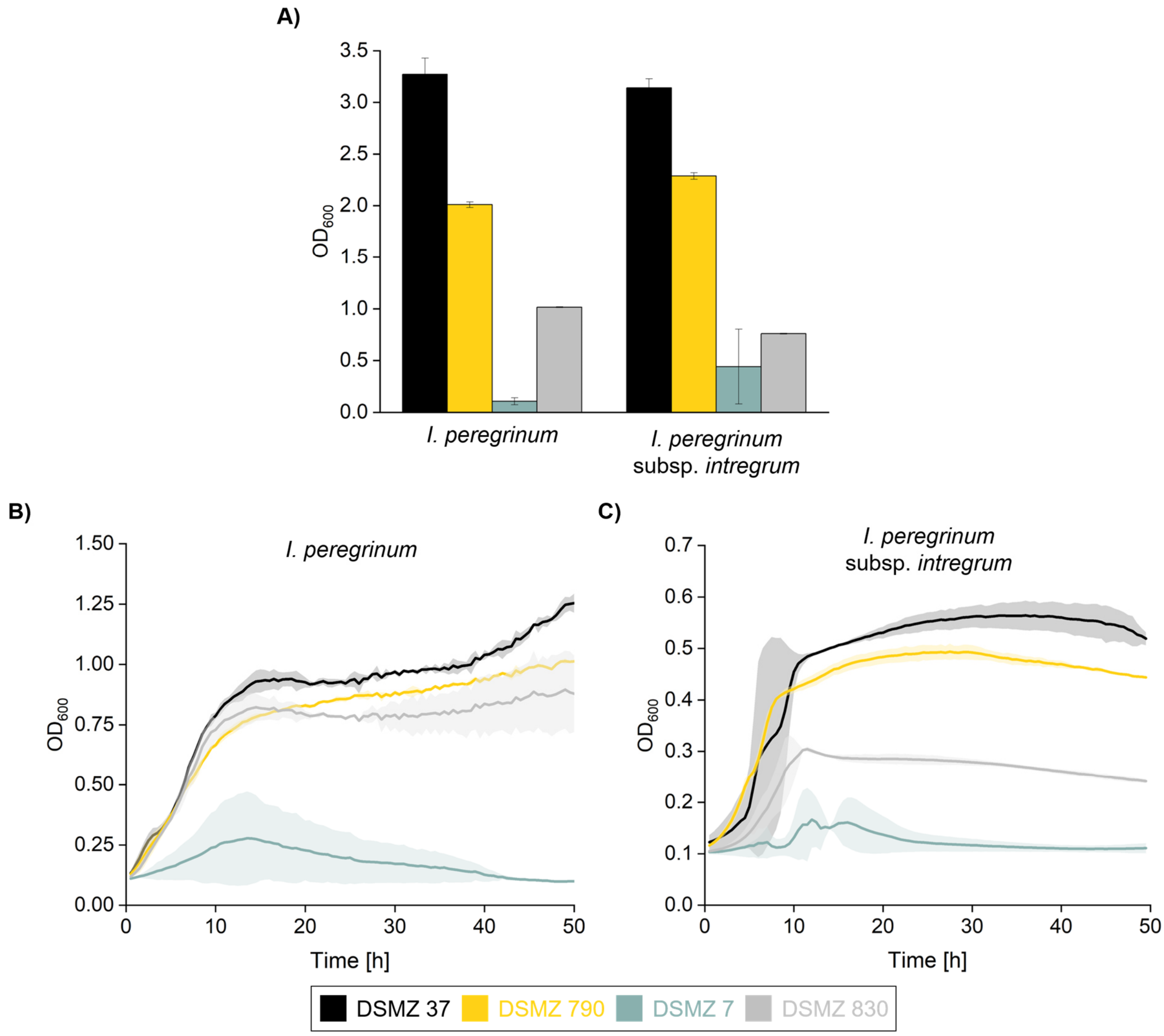

Figure S1. Growth characteristics of *I. peregrinum* and *I. peregrinum* subsp. *integrum*. Cultures were grown under atmospheric conditions at 30 °C in different media. (A) Endpoint OD_{600} values after 24 h of cultivation in Erlenmeyer flasks. (B,C) High-resolution growth curves of (B) *I. peregrinum* and (C) *I. peregrinum* subsp. *integrum* monitored over 50 h in 96-well microplates. Error bars and shaded areas represent the standard deviation of two independent experiments.

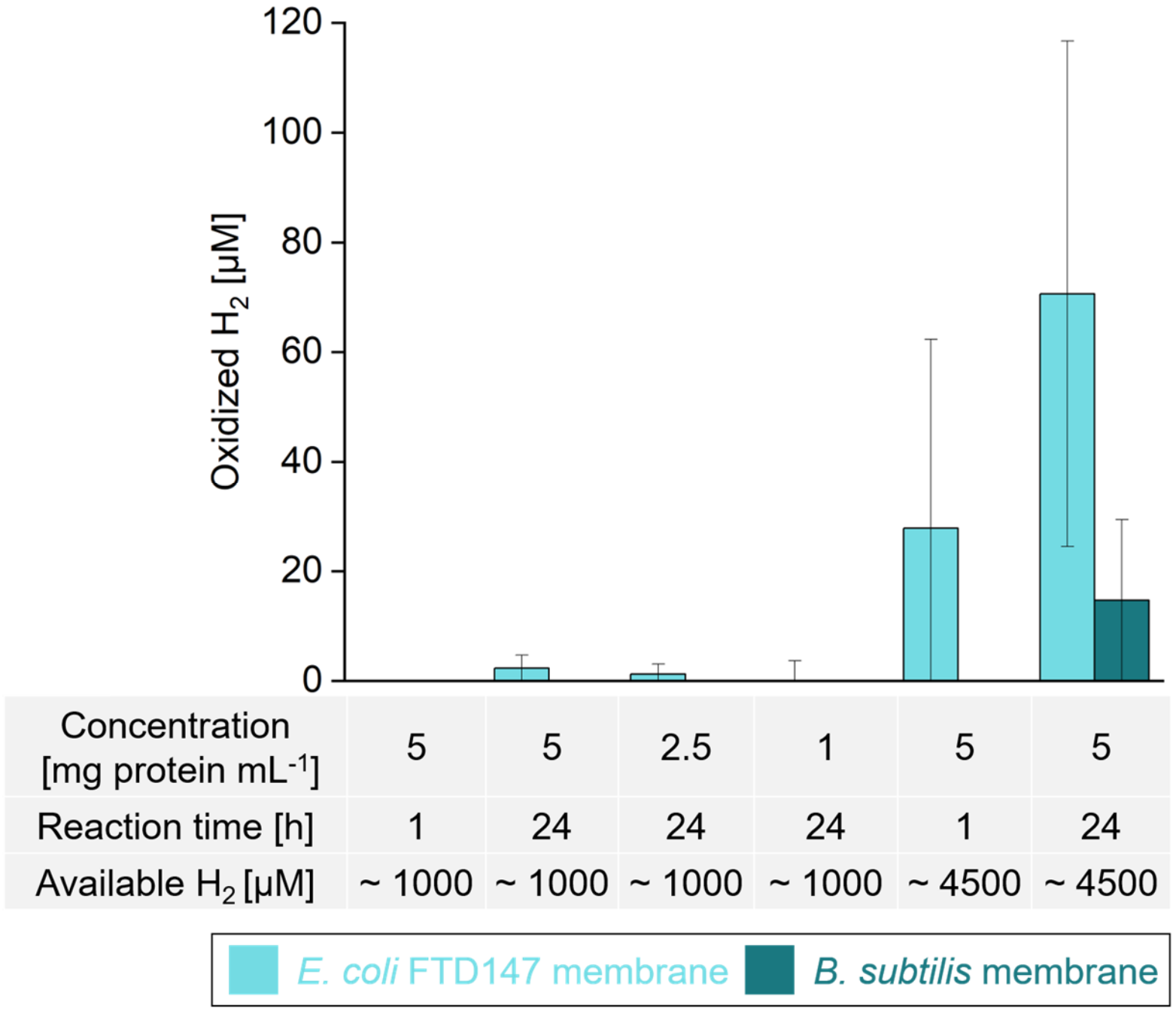

Figure S2. Hydrogen oxidation by *E. coli* FTD147 and *Bacillus subtilis* control strains. FTD147 lacks hydrogenases 1–3 but retains the *hyd-4* operon, whereas *B. subtilis* was included as an independent negative control lacking annotated hydrogenases. Assays were performed at 37 °C in 20 mL vials containing a 5 mL reaction volume and 5 mM benzyl viologen (BV) as electron acceptor. Protein concentrations, reaction times, and initial H_2 concentrations are indicated below the graph. Under standard assay conditions (~1000 µM H_2), both control strains showed negligible H_2 conversion. At an increased initial H_2 concentration of ~4500 µM, low H_2 consumption became detectable, with FTD147 consuming less than 2% of the initially available H_2 after 24 h and *B. subtilis* showing an even lower signal. Error bars represent the standard deviation of two independent experiments.

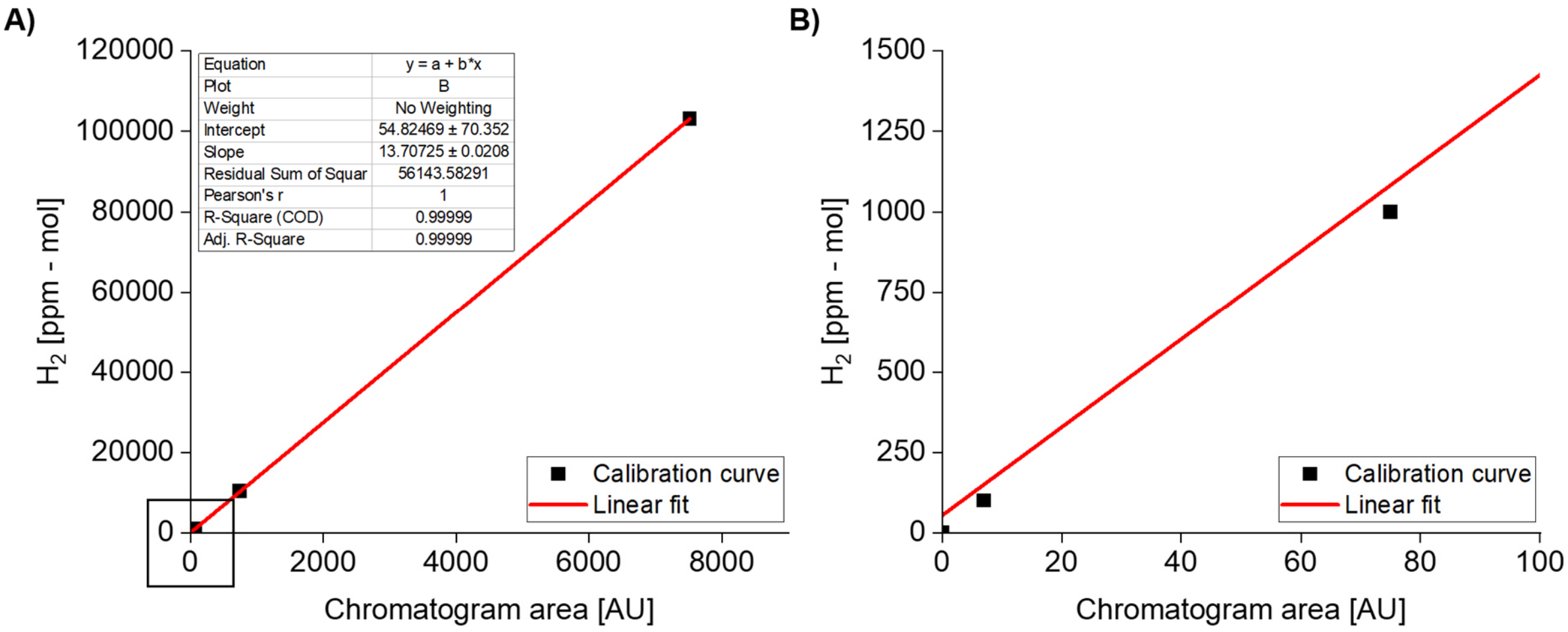

Figure S3. Calibration of H_2 quantification by gas chromatography. Gas standards containing 0, 100, 1000, 10,420, and 103,100 ppm H_2 in argon were analyzed and the corresponding chromatogram peak areas were plotted against H_2 concentration. Individual calibration data are shown as squares and the linear regression as a solid line. (A) Complete calibration range. The boxed region indicates the low-concentration range shown enlarged in (B). (B) Enlarged view of the low-concentration range using the same linear regression as in (A). Hydrogen concentrations were calculated from chromatogram peak areas using the resulting linear regression. Conversion from ppm to µM was performed by division by 22.43, corresponding to the molar gas volume under standard conditions (22.43 L mol^{-1}). The resulting equation used for quantification was: H_2 (µM) = ((peak area × 13.70725) + 54.824) / 22.43. This equation was applied to all measurements used for activity calculations.

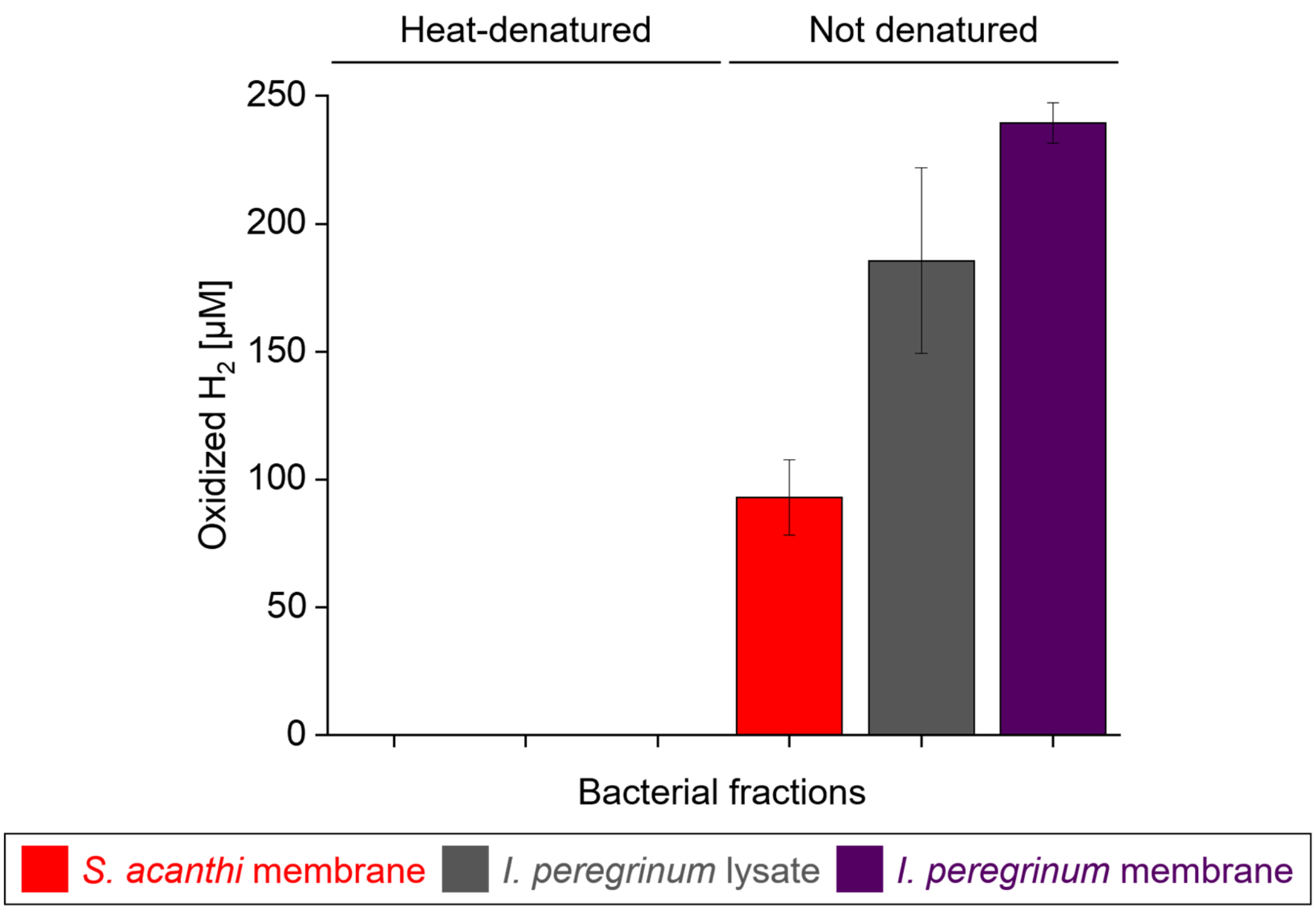

Figure S4. Effect of heat denaturation on hydrogen oxidation activity in *S. acanthi* and *I. peregrinum*. Samples were heat-denatured at 95 °C for 30 min, cooled to room temperature, and processed under standard assay conditions. No residual hydrogen oxidation activity was detected after heat treatment (left). For comparison, corresponding activities of untreated samples from Figure 2B are shown (right). Error bars represent the standard deviation of two independent experiments.

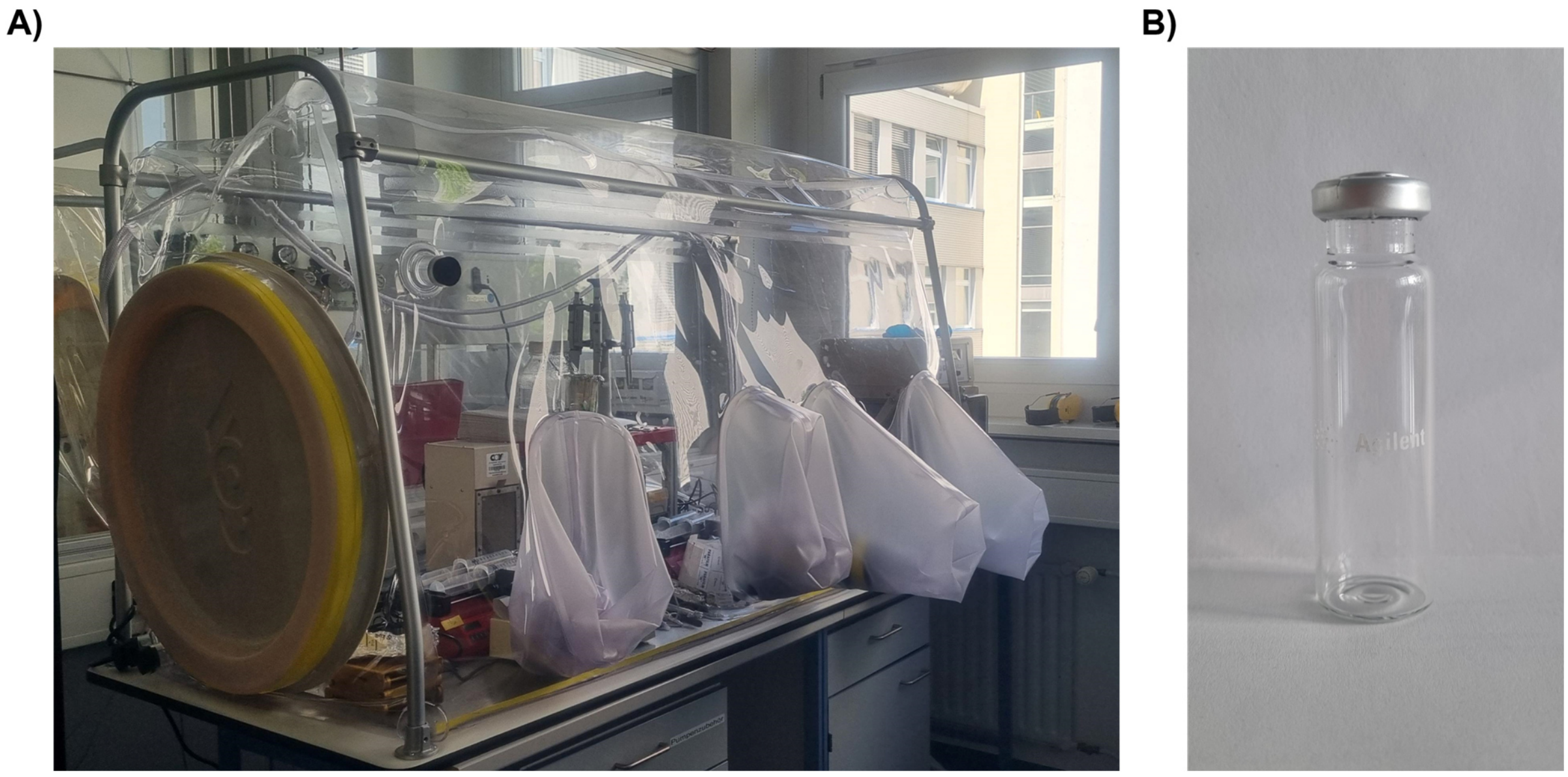

Figure S5. Experimental setup for hydrogen oxidation assays. (A) Anaerobic chamber used to establish an oxygen-free H_2/N_2 atmosphere. In this study, the chamber was used not only to remove residual O_2 but also to provide a defined hydrogen atmosphere, enabling simultaneous incubation of multiple samples under uniform conditions. (B) Representative gas chromatography (GC) vial sealed gas-tight by crimping.
